# Supplementary material for: Assessing hearing loss in older adults with a single question and person characteristics; Comparison with pure tone audiometry in the Rotterdam Study
Source: PLoS One. 2020 Jan 27;15(1):e0228349. doi: 10.1371/journal.pone.0228349 (PMC6984733; doi:10.1371/journal.pone.0228349)
Supplement: S1 Table — For 3 age categories, sex and highest achieved level of education. (DOCX) [file pone.0228349.s001.docx]

| **Educational level** | **<65 years** | | | | **65-80 years** | | | | **>80 years** | | | |
| --- | --- | --- | --- | --- | --- | --- | --- | --- | --- | --- | --- | --- |
|  | **Male** | | **Female** | | **Male** | | **Female** | | **Male** | | **Female** | |
|  | N | Mean  (SD) | N | Mean  (SD) | N | Mean  (SD) | N | Mean  (SD) | N | Mean  (SD) | N | Mean  (SD) |
| Primary | 36 | 23  (13.8) | 91 | 17  (8.3) | 74 | 27  (12.1) | 91 | 28  (19.0) | 22 | 41  (13.9) | 66 | 41  (14.7) |
| Lower | 174 | 13  (8.1) | 383 | 13  (7.9) | 252 | 28  (12.8) | 744 | 25  (11.6) | 103 | 40  (14.2) | 235 | 37  (13.9) |
| Intermediate | 276 | 17  (8.5) | 276 | 15  (8.7) | 378 | 28  (12.0) | 261 | 25  (11.9) | 142 | 41  (14.5) | 124 | 39  (12.9) |
| Higher | 276 | 15  (8.3) | 260 | 14  (9.4) | 312 | 26  (12.2) | 167 | 21  (9.1) | 81 | 35  (13.1) | 35 | 35  (13.9) |
