# Supplementary material for: Assessing hearing loss in older adults with a single question and person characteristics; Comparison with pure tone audiometry in the Rotterdam Study
Source: PLoS One. 2020 Jan 27;15(1):e0228349. doi: 10.1371/journal.pone.0228349 (PMC6984733; doi:10.1371/journal.pone.0228349)
Supplement: Attachment — Submitted filename: Response to the reviewers.docx [file pone.0228349.s003.docx]

Reviewer #1: Authors have addressed all of my concerns. I believe the document should be published. I congratulate them on an interesting paper.

**Response:** *We thank the reviewer for his/her work.*

Reviewer #2: Thanks for addressing the reviewer concerns. The paper may still benefit from some editorial overview.

**Response:** *We thank the reviewer for his/her work and input, we have carefully gone through the text again.*

Reviewer #3: The authors have responded reasonably to my comments. I still feel that there is a tendency to overclaim the effectiveness of the "single question' in identifying hearing loss. In the author's response to one of my comments, it is noted that age, gender and education have high predictability for hearing loss (without the question) with an AUC (area under the ROC curve) of 0.82. This does improve to 0.86 with the use of the question but it suggests that the prediction is not greatly improved. i feel this information should be included in the paper.

**Response:** *We thank the reviewer for his work. We agree this is not an huge increase, however the scope of our paper is to investigate the question as the main outcome. The additional information in this paper is that the ability to identify hearing loss can be improved when age is taken into account on a population level.*

Reviewer #4: A clean copy (not showing changes) has not been provided; the initially submitted version was instead. Furthermore, there were two versions of the figures.
The authors appear to have tackled all the comments and suggestions that I and the other reviewers made. However, I suggest another careful review of the English a few errors in wording and sentence structure are still there.
**Response:** *We thank the reviewer for his careful review of the manuscript. We hope that we have sufficiently addressed the issues raised by the reviewer.*

Line 77: capital S

**Response:** *We have changed this.*

Line 79: Not sure this captures the matter correctly. And the sentence swaps from the prevalence of hearing loss in younger adults (population) to their own hearing (an individual). I agree that ease of estimation is judged on a personal level. But the wording suggests that it is easier to estimate good hearing than poor(er) hearing. Is that the case? And that the ease translates to better estimation of prevalence. This section needs some work. I can see what the authors are getting at, but I am not convinced. And is this not complicated with the issue noted in the Discussion in Lines 267 onwards?
**Response:** *We thank the reviewer for this point. What we aim to point out here is that statistics of self-reported hearing loss in a younger group is highly influenced by the relatively low prevalence of hearing loss, and should not be compared with the statistics of an older population. As this point has been addressed in more detail in the discussion, we shortened and rephrased the text in this section.*

Line 84: Starting with ‘While’ leads the reader to expect more in the sentence. I suggest dropping the word.

**Response:** *We have dropped the word while.*

Line 89: ‘have’ is correct, not ‘has’

**Response:** *We have changed has to have.*

Line 121: I know that for the previous review it was not necessary to point out that the question and answers were in Dutch. However, upon reflection it would be good to make that clear, in case someone uses the same wording, and then compares results; this would not be entirely appropriate as the English translation/version has not been validated.

**Response:** *We have added to the main text that the question was in Dutch.*

Line 133: is it really not possible to reference this?

**Response:** *Unfortunately not. This is done in all studies to be able to calculate with the hearing thresholds when the threshold of a participant is above the maximum stimulation level.*

Table 1: The age range also needs to have ‘years’ in the first column

**Response:** *We have added this to the table.*

Line 177: ‘Hearing thresholds were’, not ‘Hearing loss was’

**Response:** *We have changed this.*

Line 181: ‘thresholds’? But actually it’s PTA.5-4 isn’t it?

**Response:** *We have changed the sentence to ‘…increase of the PTA_0.5-4_ hearing threshold…’ as we feel this reflects the result best and meets the suggestion of the reviewer.*

Lines 188 to 195; two versions of the table caption.

**Response:** *We see what the reviewer means, but the second capitation is for figure 2.*

Table 3: The placement of the words ‘Prevalence’ is a bit unhelpful when the 100% figures are seen. Can these 100% figures be removed?

**Response:** *We have removed the 100% figure.*

Line 240: This needs to be more nuanced. To start with, I do not think anyone would think it would replace clinical assessment of individuals, which this implies. On the other hand, I think it may have some clinical value in identifying people who are at a higher risk of hearing loss, and so this information has potential for use as a screening tool.

**Response:** *We agree with the reviewer there are different perspectives on how the results can be interpreted. Therefore, we have chosen to emphasize the value of this tool in population based studies. The statement about the clinical value has been rephrased in a way that the perspective of screening has been taken in account, according to the reviewers comment.*

Line 259 onwards: Comment/question: were males better or worse at estimation than females? This matter of self-reporting is complicated by things like denial of hearing problems and self-awareness which may be lower in males, but on the other hand males having a higher prevalence of HL. I think the authors have grappled with some of these issues.

**Response:** *Males were better at the estimation than females, in spite of many other possible factors we think that the higher prevalence of hearing loss in males might have played an important role explaining this difference.*

Line 266: points?

**Response:** *We have changed point to points.*
